# An unusual presentation of carcinoma stomach

**DOI:** 10.11604/pamj.2013.14.84.1203

**Published:** 2013-03-05

**Authors:** Jeffey George, Deepak Kochummen Johnson, Rajneesh Anugraha Rajan, Sandesh Kolassery, Ramachandran Mavali Thazhath

**Affiliations:** 1 Department of gastroenterology, medical college, Calicut, India

**Keywords:** Adenocarcinoma stomach, dic, bone metastasis

## Abstract

Symptomatic gastric malignancy usually presents with symptoms which mimic peptic ulcer disease.Usual presenting features include weight loss and abdominal pain. Other symptoms include nausea, vomiting, dysphagia, melena and early satiety. Gastric malignancy presenting with hemetemesis, macular skin lesions of DIC and low backache due to bone metastasis from the primary is rare. Also bone metastasis in gastric cancer in the absence of hepatic metastasis is also rare.

## Introduction

Gastric malignancy can have protean manifestations. We present a rare manifestation of adenocarcinoma of stomach in a young patient.

## Patient and observation

A 33-year man working in Gulf, presented with hematemesis 15 days ago in the month of February this year. This was preceded by low grade fever, bone pain, generalised body ache, low back ache and generalised macular skin rashes. There was no history of abdominal pain, jaundice or features of chronic liver disease.He gave history of intake of analgesics for backache. After symptomatic treatment he returned back. He came to our OPD with Complete blood count which showed a haemoglobin of 8g/dl, total count 6700/mm3 platelet count 24000/mm3 and ESR 55mm/hr. On examination patient was pale with non-pruritic macular rashes and mild splenomegaly.Residing in Middle East with background history of fever, backache, skin rash, splenomegaly and a low platelet count possibility of atypical infection like Brucellosis with NSAID induced gi bleed was considered.

**Figure 1:**
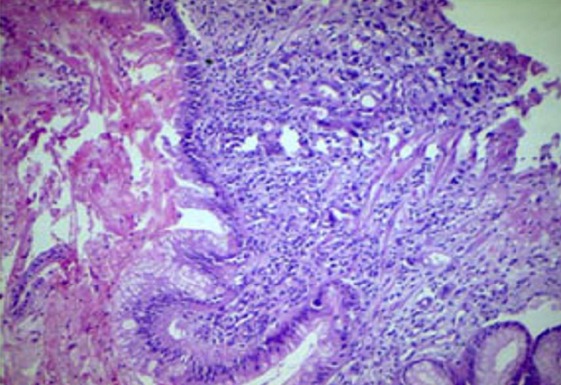
Adenocarcinoma stomach (above) low power showing tumor cells diffusely and a vague glandular pattern composed of cells with hyperchromatic and pleomorphic nuclei and prominent nucleoli

**Figure 2:**
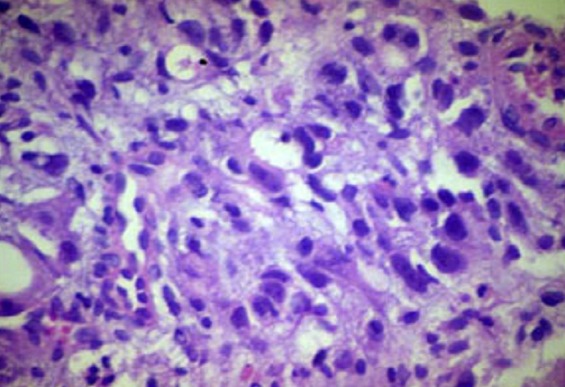
Adenocarcinoma stomach (below) high power showing glands lined by cells having pleomorphic vesicular nuclei with prominent nucleoli

**Figure 3:**
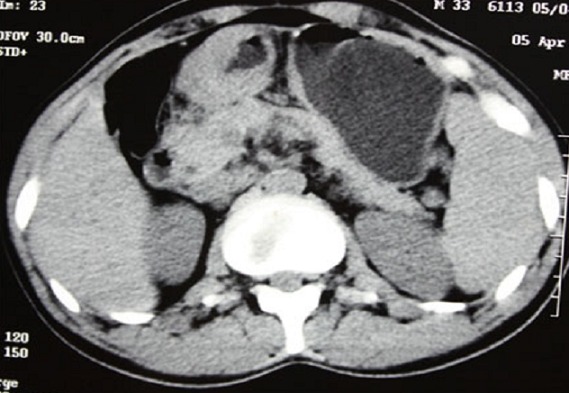
Adenocarcinoma stomach -CT scan of abdomen showing gross thickening of pylorus with regional lymphadenopathy suggestive of advanced gastric malignancy

**Figure 4:**
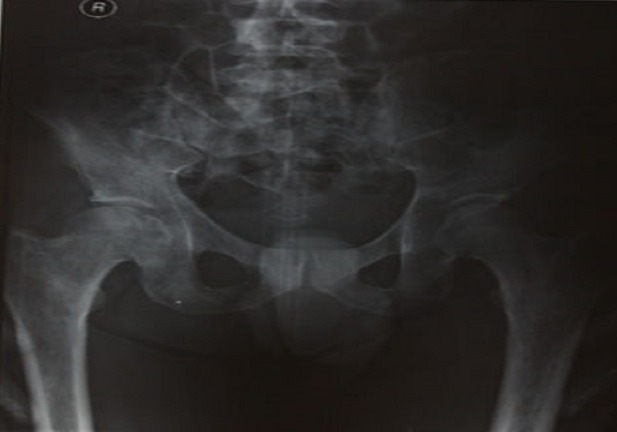
X-ray AP pelvis showing lytic bone lesions in the right femur

He was admitted for further evaluation and endoscopy done showed a nodular ulcerative lesions in the antrum of stomach and histopathologic examination confirmed adenocarcinoma of stomach (Figure 1, Figure 2, Figure 3). Liver function tests showed a high ALP (1536 U/l), elevated INR (2.15) though bilirubin, transaminases and serum proteins were normal. Brucella agglutination test was negative. Xray Chest was normal. Ultrasound of abdomen showed normal echo texture of liver, no SOL, normal portal vein and no IHBRD. CT scan of the abdomen revealed gross pylorus thickening with regional lymphadenopathy and skeletal metastasis with sclerotic foci involving vertebrae and pelvic bones and lytic areas in bilateral iliac blades with no liver metastasis or ascites. A skeletal survey was done which showed lytic lesions in the iliac and femoral bones. Bone marrow aspirate showed decreased red cells and platelets and marrow biopsy done was reported as metastasis from adenocarcinoma (Figure 4). Coagulation profile done was suggestive of DIC due to the raised levels of FDP (5,170 ng/dl) and a low fibrinogen (152mg/dl).

He was transferred to Medical Oncology department for subsequent management and was treated with chemotherapy. He succumbed to his illness within 10 months of diagnosis.

## Discussion

This young patient had adenocarcinoma stomach with bone metastasis, marrow infiltration and DIC in the absence of liver metastasis. Bone metastasis are classically associated with carcinoma of thyroid, kidney, lung and prostrate. Bone metastasis occurs in only 0-17% of cases of gastric carcinoma [1] which can be either osteosclerotic or osteolytic [2]. In this case there was no involvement of liver which points to a non portal spread of the tumor probably via Batsons plexus [3] which explains the distant metastasis in the absence of liver metastasis. Apart from bone metastasis the patient also had evidence of Disseminated Intra Vascular Coagulation.The incidence of DIC ranges from 10 - 75% [4]. DICobserved in advanced cancer patients is due to acute or gradual development of fibrin microthrombi which may be due to multiple factors such as direct endothelial injury, coagulation cascade activation, production of procoagulant substances and reduction in the synthesis or activity of anticoagulant factors. Even though coagulation abnormality can occur in carcinoma stomach due to various factors clinically manifest DIC as in this patient is rare [5, 6] Reports of adenocarcinoma stomach presenting as DIC are usually associated with large tumor burden and metastasis [4].

## Conclusion

Gastric neoplasm should be considered in the differential diagnosis of metastatic bone lesions with unknown primary. Rarely DIC can be the presenting feature of stomach malignancy.
